# D-Dimer in Viral Respiratory Infections: Established Evidence in COVID-19 and Emerging Evidence in Other Respiratory Viruses

**DOI:** 10.3390/ijms27198525

**Published:** 2026-09-24

**Authors:** Yahya Madkhali, Ayman Mubarak, Bandar Alosaimi, Wael Alturaiki

**Affiliations:** 1 Department of Medical Laboratory Sciences, College of Applied Medical Sciences, Majmaah University, Majmaah 11952, Saudi Arabia; 2 Department of Botany and Microbiology, College of Science, King Saud University, Riyadh 11451, Saudi Arabia; 3 Research Center, King Fahad Medical City, Riyadh Second Health Cluster, Riyadh 11525, Saudi Arabia

**Keywords:** D-dimer, viral respiratory infections, prognostic biomarker, endothelial dysfunction, immunothrombosis, risk stratification, narrative review, evidence mapping

## Abstract

Viral respiratory infections remain a major cause of global morbidity and mortality, with severe disease increasingly recognized to involve profound interactions between inflammation, endothelial dysfunction, coagulation activation, and immunothrombosis. D-dimer, a fibrin degradation product and established biomarker of fibrin turnover, has gained considerable attention because of its strong association with adverse outcomes in coronavirus disease 2019 (COVID-19). This narrative review compares the evidence for D-dimer in COVID-19 with that available for other respiratory viruses and deliberately separates three questions that are frequently conflated: whether D-dimer predicts severity or death (prognostic), whether it identifies or excludes venous thromboembolism (diagnostic), and whether it identifies patients who benefit from anticoagulation (treatment-predictive). In COVID-19, elevated D-dimer is reproducibly associated with disease severity, thromboembolic complications, intensive care unit admission, and mortality, and the association persists after adjustment; however, discrimination is only moderate, incremental value over routine clinical variables has rarely been tested, and randomized trials have not established D-dimer as a treatment-selection biomarker. Outside of COVID-19, the evidence is substantially weaker and uneven: influenza is supported mainly by comparative cohorts, adenovirus by a single unvalidated paediatric prediction model, and the only direct study of D-dimer and clinical severity in bronchiolitis was negative, while for MERS-CoV, parainfluenza, and human metapneumovirus, we identified no study relating D-dimer to a clinical outcome. Moreover, substantial heterogeneity in assay methodology, sampling strategies, and proposed cut-off values restricts standardization and cross-study comparisons. We therefore present COVID-19 as a mechanistic and methodological reference rather than a validated universal rule, and summarize the evidence in a structured table and a visual evidence map. Overall, D-dimer appears to represent an integrated biomarker of coagulation activation, endothelial injury, and systemic inflammation rather than thrombosis alone, supporting its role in risk stratification while underscoring the need for context-specific interpretation. The cross-viral propositions advanced here should be regarded as hypothesis-generating. Future prospective multicentre studies using standardized assays, serial biomarker assessment, and multimarker prediction models are required to establish pathogen-specific prognostic frameworks and define the role of D-dimer in precision medicine for viral respiratory infections.

## 1. Introduction

Viral respiratory infections remain a major cause of global morbidity and mortality, and continue to impose substantial pressure on health systems across all age groups. In 2021 alone, non-COVID-19 lower respiratory infections accounted for an estimated 344 million incident episodes and 2.18 million deaths worldwide, while influenza, respiratory syncytial virus (RSV), COVID-19, and other epidemic-prone respiratory viruses continue to shape seasonal and outbreak-related severe disease burdens [1,2,3].

D-dimer, a degradation product of cross-linked fibrin, is an established biomarker of coagulation activation and fibrinolysis and is widely used in thrombotic disorders [4,5]. Its relevance in viral respiratory infections has gained increasing attention because these infections can trigger a complex host response characterized by systemic inflammation, endothelial injury, platelet activation, tissue factor induction, and immunothrombosis, all of which promote fibrin formation and subsequent fibrin breakdown [6,7,8]. In severe COVID-19, histopathologic and mechanistic studies have linked endothelial dysfunction and microvascular thrombosis to organ injury, while hypoxia and inflammatory cell infiltration appear to further amplify a procoagulant milieu [7,9].

Current clinical evidence indicates that elevated D-dimer is consistently associated with adverse outcomes in hospitalized patients with COVID-19, including severe disease, intensive care requirement, venous thromboembolism, and mortality [10,11]. Admission D-dimer values of approximately 2.0 μg/mL have been reported to discriminate patients at increased risk of in-hospital death, and rising D-dimer levels during hospitalization appear to provide additional prognostic information [4,12]. However, this evidence base is heavily weighted toward COVID-19. By contrast, data for influenza A, RSV, MERS, SARS, and other respiratory viruses are comparatively sparse, often indirect, and fragmented across small observational studies or mechanistic reports, although available findings suggest similar links between coagulation activation, thrombosis, and poor outcomes in at least some non-COVID-19 viral infections [8,11]. In this review, we treat that apparent similarity as a hypothesis to be tested rather than as an established finding.

A central unresolved question is whether D-dimer primarily reflects thrombotic risk, inflammatory burden, or overall disease severity across different viral respiratory infections. Addressing this uncertainty requires comparative evaluation across viruses with distinct tropisms, inflammatory phenotypes, and thrombotic profiles. This narrative review therefore examines the prognostic significance of D-dimer beyond COVID-19, with the aim of comparing available evidence across major viral respiratory infections, identifying shared and virus-specific patterns, and highlighting priorities for future research. Because the non-COVID-19 evidence base is small and fragmented, the comparative propositions developed here are explicitly hypothesis-generating rather than confirmatory, and the title of this review has been framed accordingly.

Three distinct questions are frequently conflated in the literature, and we keep them separate throughout. The first is prognostic: does D-dimer predict disease severity, organ support, or death? The second is diagnostic: does D-dimer identify or exclude venous thromboembolism (VTE) in a patient already known to be infected? The third is treatment-predictive: does D-dimer identify patients who benefit from escalated anticoagulation? A biomarker may perform well on one of these and poorly on the others, and evidence for one does not transfer to another. Within the prognostic question, we further distinguish four ascending levels of claim: (i) an unadjusted association with outcome; (ii) an independent association after adjustment for illness severity and comorbidity; (iii) incremental discrimination beyond routine clinical variables; and (iv) demonstrated improvement in patient management or outcome. Almost all available evidence, including in COVID-19, sits at levels (i) and (ii), and we indicate throughout which level a given finding supports.

### Methods of Literature Search

A non-systematic, narrative literature search was performed in PubMed/MEDLINE, Scopus, and Google Scholar for articles published between January 2000 and August 2026 (final search run on 20 August 2026, updated from the original submission), restricted to English-language full texts. Search terms combined “D-dimer,” “coagulopathy,” “biomarker,” “prognosis,” “severity,” “mortality,” and “immunothrombosis” with pathogen-specific terms (“COVID-19,” “influenza,” “RSV,” “SARS,” “MERS,” “adenovirus,” “parainfluenza,” “human metapneumovirus”), and reference lists of retrieved articles were hand-searched for additional relevant studies. The complete search strings used for each database and each pathogen are provided in Supplementary Table S1.

Original research, cohort studies, systematic reviews, meta-analyses, and mechanistic studies addressing D-dimer in relation to disease severity, ICU admission, thromboembolism, or mortality in viral respiratory infection were considered. As this is a narrative review, no protocol was registered and PRISMA reporting was not applied; reporting instead follows the SANRA quality standard for narrative reviews. Study selection and synthesis relied on relevance and quality, prioritizing large studies and meta-analyses where available, with smaller or mechanistic studies used to illustrate biological plausibility for less-studied viruses. To make the evidence base auditable, we then extracted for each pathogen, where reported, the study design and sample size, the D-dimer assay and reporting units, the outcome assessed, whether the reported association was adjusted, and any proposed threshold with its discrimination statistics. These are tabulated in Table 1 together with the number of primary clinical studies linking D-dimer to a clinical outcome that we were able to identify for each virus. Because pathogen-specific yields outside COVID-19 and influenza were extremely small, we report the number of eligible studies retained rather than full screening counts, and we state explicitly where no eligible study was identified rather than substituting indirect evidence. Findings were synthesized qualitatively and organized thematically rather than pooled quantitatively. No new patient-level data were generated or analysed. This approach has clear limitations: the search was non-systematic and restricted to English-language reports, it is subject to the same publication bias that has favoured COVID-19, and the evidence grades presented in Table 1 and Figure 1 are structured but ultimately reflect author judgement rather than formal risk-of-bias assessment.

## 2. Pathophysiological Basis of D-Dimer Elevation

D-dimer elevation in viral respiratory infections reflects overlapping coagulation, endothelial, and immune pathways rather than a single mechanism, and the balance among these pathways appears to differ by virus and by disease severity. Across COVID-19, influenza, SARS/MERS, and severe viral pneumonias more broadly, the most consistent pattern is that higher D-dimer accompanies more severe lung injury and worse outcomes, but the biological meaning of that signal remains partly unresolved [36,37] (Figure 2).

### 2.1. Coagulation Activation During Viral Infection

Direct activation of coagulation is one plausible source of D-dimer elevation, but the evidence suggests it is rarely an isolated process. Across pathogenic coronaviruses and influenza A, viral infection induces tissue factor expression in lung and circulating immune compartments, and this is accompanied by extracellular vesicle-associated procoagulant activity that correlates with D-dimer, thrombosis, severity, and mortality in severe disease [8]. This cross-virus convergence argues that fibrin formation is biologically real, not merely an epiphenomenon of inflammation.

At the same time, elevated D-dimer should not be equated too simply with classic disseminated intravascular coagulation. In severe COVID-19, hypercoagulability often coexists with only mild thrombocytopenia, limited PT prolongation, and preserved fibrinogen early in the course, a pattern distinct from conventional septic DIC and more consistent with localized or compartmentalized thrombin generation, particularly in the lung [36]. Influenza can also progress to DIC in the setting of ARDS or multiorgan failure, but comparative work suggests that similarly high D-dimer levels can arise in both influenza and COVID-19 despite broader coagulation abnormalities in COVID-19, implying partially shared but non-identical hemostatic phenotypes [19].

The major limitation of the literature is that mechanistic inference is often drawn from biomarker associations, animal models, or severe hospitalized cohorts. Human challenge data hint that even mild respiratory viral infection can increase thrombotic biomarkers, but the pattern differs: SARS-CoV-2 broadened platelet/thrombotic activation markers, whereas H3N2 produced a later isolated D-dimer rise without measurable endothelial biomarker changes [23]. This suggests that D-dimer can rise even when overt endothelial dysfunction is not captured in plasma, complicating any single-pathway interpretation.

### 2.2. Endothelial Dysfunction and Immunothrombosis

Endothelial injury provides a stronger explanation for why D-dimer tracks severity so consistently in severe respiratory viral disease. In COVID-19, markedly increased von Willebrand factor and factor VIII support an endothelial-activated procoagulant state, while direct or complement-mediated endothelial injury has been proposed to promote release of ultra-large vWF multimers, platelet recruitment, and microthrombogenesis [7]. Reviews comparing severe SARS-CoV-2 with other coronaviruses and influenza place endothelial dysfunction within a broader immunothrombotic framework rather than as an isolated vascular lesion [6,38].

This framework is useful because it explains why D-dimer may reflect both thrombosis and host defense. Immunothrombosis links activated neutrophils, monocytes, platelets, and coagulation into an intravascular containment response that becomes pathological when excessive, especially in the pulmonary microcirculation [6,36]. Experimental and translational data from MERS, SARS, influenza, and COVID-19 all support pulmonary microthrombus formation, but the strength of evidence varies considerably, being richest for COVID-19 and much thinner for RSV and non-coronavirus respiratory viruses [8,26].

A key unresolved issue is whether elevated D-dimer primarily indicates active intravascular thrombosis or downstream fibrin turnover from injured lung tissue. That distinction matters clinically because pulmonary fibrin deposition, alveolar-capillary leak, and local fibrinolysis could generate high D-dimer even without macrovascular venous thromboembolism. The available evidence supports both interpretations, which likely coexist rather than compete [36].

### 2.3. Interaction Between Inflammation and Coagulation

The strongest consensus across respiratory viruses is that inflammation and coagulation amplify one another. Viral infection disrupts the balance between procoagulant and anticoagulant pathways through cytokine release, leukocyte and platelet activation, endothelial perturbation, and suppression of fibrinolytic control [26]. In severe COVID-19, this interaction has often been framed as thrombo-inflammation rather than pure thrombosis, and that framing is useful beyond COVID-19 because influenza shows similar cytokine-driven platelet activation and immune-cell aggregation, albeit with subtype-dependent intensity [19,39].

Yet, inflammation alone does not fully explain D-dimer behavior. In COVID-19, D-dimer correlates with inflammatory markers and often falls as inflammation resolves, but patients can still show disproportionately high D-dimer compared with bacterial pneumonia at similar CRP ranges, implying additional virus-specific or lung-compartment mechanisms [40]. Platelet studies add further nuance: platelets are hyperactivated and can contribute to cytokine release, but D-dimer does not correlate tightly with every platelet activation marker, arguing against a simple one-biomarker surrogate model of thrombo-inflammation [41].

The fibrinolytic literature reinforces this ambiguity. Very high D-dimer may signal inefficient clot clearance, but a failing attempt to remove fibrin-rich and necrotic material from damaged lung parenchyma, with timing determining whether plasmin activity is adaptive or harmful [42]. Thus, D-dimer should be interpreted less as a specific marker of one event than as an integrated readout of fibrin formation and breakdown within an inflammatory organ injury state.

### 2.4. Mechanisms Unique to Severe Respiratory Viral Diseases

Severe disease likely produces higher D-dimer because it concentrates several processes that are modest or transient in mild infection: greater viral burden, more diffuse alveolar injury, stronger cytokine signaling, broader endothelial activation, increased platelet–leukocyte interactions, and more extensive pulmonary microvascular fibrin deposition. Meta-analytic evidence in COVID-19 shows that D-dimer and fibrinogen are the hemostatic variables most strongly associated with severity, supporting the view that D-dimer is not a random abnormality but part of the biology of progression [37].

However, severe respiratory viral disease is not mechanistically uniform. SARS-CoV appears to affect fibrinolytic balance through PAI-1 and urokinase-pathway dysregulation, influenza engages plasminogen-dependent inflammatory injury, and SARS-CoV-2 combines these shared pathways with particularly prominent endothelial activation and platelet hyperreactivity [41,43]. For RSV and several other common respiratory viruses, direct mechanistic evidence remains sparse, so cross-virus generalizations still rest heavily on extrapolation from COVID-19, influenza, and prior epidemic coronaviruses.

Overall, current evidence suggests that D-dimer elevation is best understood as the convergence of thrombosis, endothelial dysfunction, fibrinolysis, and immune dysregulation, with the relative weight of each mechanism shifting across viruses and across stages of illness. That mechanistic heterogeneity is precisely why the same laboratory abnormality can indicate active thrombosis in one patient, predominant inflammatory lung injury in another, and a composite severity signal in both. The next section therefore considers how this biologic complexity shapes the prognostic interpretation of D-dimer across viral respiratory infections.

## 3. Evidence Across Major Viral Respiratory Infections

Before considering each pathogen in turn, Table 1 summarizes the clinical evidence base linking D-dimer to outcome for each virus, and makes explicit where evidence is absent rather than merely weak. The asymmetry is the central observation of this review: the COVID-19 column is populated at every level of claim, whereas most other viruses are supported only by mechanistic plausibility.

### 3.1. SARS-CoV-2

COVID-19 is included here as the reference case rather than as the subject of this review, and is summarized briefly because it has already been reviewed extensively elsewhere. It combined three features rarely documented so clearly in earlier respiratory viral infections: frequent D-dimer elevation, an unusually visible thrombotic phenotype, and large hospital cohorts that linked this laboratory abnormality to clinically important outcomes. Higher admission or peak D-dimer tracks with worsening clinical severity, critical illness, and death across both unadjusted and adjusted analyses [10,17,44]. At the level of association, and of independent association after adjustment, the COVID-19 evidence is therefore strong.

The higher levels of claim are far less secure. Thresholds around 1.5–2.0 mg/L often perform reasonably well, but reported cut-offs vary by assay, timing, outcome definition, and case mix, and when admission values were harmonized across a large multicentre registry, D-dimer remained independently associated with mortality while discriminating only modestly, with an AUC of 0.66 [5,13,14]. Whether D-dimer adds discrimination beyond routine clinical variables such as age, oxygenation, and comorbidity has rarely been tested formally, even in COVID-19.

The link with thrombosis is more convincing in COVID-19 than in other viral pneumonias, but it is a diagnostic rather than a prognostic claim. Critically ill COVID-19 cohorts showed substantial thrombotic complication rates even under prophylactic anticoagulation, and very high admission D-dimer was independently associated with subsequent coagulation-related complications [15,16]. Even so, D-dimer behaves as a high-sensitivity marker of thrombo-inflammatory risk rather than a specific indicator of macrovascular venous thromboembolism: elevations also track C-reactive protein, fibrinogen, and endothelial activation, and remain associated with mortality and radiographic severity in cohorts where deep vein thrombosis and pulmonary embolism were actively excluded [13,16,45,46]; the resulting syndrome overlaps with, but is not identical to, disseminated intravascular coagulation [47,48]. Consensus statements accordingly support measuring D-dimer to assess severity and thrombotic risk while discouraging its use alone to escalate anticoagulation [10,46,49]. COVID-19 therefore supplies a strong association, a moderate diagnostic signal, and no established treatment-predictive role, and this is the ceiling against which the remaining viruses should be judged.

### 3.2. Influenza

Influenza provides the most important comparator to COVID-19 because both are severe viral pneumonias capable of activating coagulation pathways, yet the clinical prominence of D-dimer has been far lower in influenza. Available comparative evidence suggests that elevated D-dimer is not unique to COVID-19; in one direct cohort comparison, D-dimer levels were similarly high in hospitalized influenza and COVID-19 patients, even though COVID-19 showed a more refractory and platelet-driven coagulopathy profile overall [19]. This is an important corrective to the assumption that any D-dimer elevation in viral pneumonia implies a COVID-19-like thrombotic syndrome. A small single-centre prospective study that measured D-dimer in parallel across influenza A pneumonia, COVID-19, septic shock, and pulmonary embolism in critically ill adults, and whose patient-level data are openly deposited, provides one of the very few directly harmonized comparisons and reinforces that high D-dimer in critical illness is not virus-specific [20].

The difference is therefore not simply whether D-dimer rises, but what that rise means prognostically. In influenza, coagulation abnormalities appear biologically plausible and mechanistically linked to immune-mediated lung injury, but the modern evidence base does not demonstrate the same consistent, independently validated association with thrombosis, ICU requirement, and mortality that exists for COVID-19 [5,13,26]. Part of the explanation lies in the outcome rather than in the biomarker. Thrombotic events are genuinely less frequent in influenza: in a Dutch cohort, the 30-day cumulative incidence of any thrombotic complication was 11% among 13,217 hospitalized influenza patients versus 25% in COVID-19 [24]; in the United States Sentinel System, the 90-day absolute risk of venous thromboembolism was 5.3% after influenza hospitalization versus 9.5% and 10.9% after COVID-19 hospitalization before and during vaccine availability, with adjusted hazard ratios of 1.60 and 1.89, respectively [25]. A lower event rate lowers the positive predictive value of any given threshold, so a cut-off imported from COVID-19 would be expected to perform worse in influenza even if the underlying biology were identical. Influenza-associated D-dimer elevation may mark severe systemic illness, but current studies do not establish it as a robust stand-alone prognostic biomarker ready for routine clinical deployment.

This distinction likely reflects both biology and evidence architecture. COVID-19 produced a conspicuous endothelial and thrombo-inflammatory phenotype that prompted systematic coagulation testing and outcome-focused analyses at scale, whereas influenza cohorts were less often designed around thrombosis biomarkers, and D-dimer was historically not incorporated into routine prognostic algorithms for viral pneumonia [5,46]. As a result, extrapolating COVID-19-derived D-dimer thresholds to influenza is not currently justified. The available evidence supports biological relevance, but not routine influenza-specific prognostic use. We identified no study testing whether D-dimer identifies influenza patients who benefit from anticoagulation, so the treatment-predictive question is unaddressed rather than answered negatively.

### 3.3. Respiratory Syncytial Virus (RSV)

RSV illustrates the gap between mechanistic plausibility and clinical evidence more sharply than any other virus considered here. Severe RSV infection induces a robust inflammatory response characterized by cytokine release, endothelial activation, and increased vascular permeability, all of which promote tissue factor-mediated coagulation and subsequent fibrin degradation, so elevated circulating D-dimer is biologically expected as a downstream consequence of immunothrombosis within the pulmonary microvasculature [35]. That expectation, however, is inference from the general pneumonia and COVID-19 literature rather than a finding derived from RSV-specific data, and the two should not be presented as equivalent.

Direct evidence is very limited and, to date, negative. In the only study we identified that tested D-dimer against clinical outcome in bronchiolitis—the syndrome for which RSV is the predominant cause—102 hospitalized infants and young children showed no significant relationship between serum D-dimer and clinical severity score (*p* = 0.53), length of hospital stay (*p* = 0.79), or admission to paediatric intensive care (*p* = 0.48), and no deaths occurred [27]. That study was retrospective and single-centre, patients with COVID-19 and with pre-existing thrombotic or cardiac disease were excluded, and the overall clinical course was mild, so it cannot exclude an association in more severe disease or in adults. It is nonetheless the only direct test available, and it did not support one. Recent reviews of RSV pathogenesis and management discuss severity biomarkers without establishing coagulation markers as validated prognostic tools [28].

Two further considerations limit what can reasonably be claimed. D-dimer concentration is influenced by age, bacterial co-infection, chronic cardiovascular disease, thromboembolic disorders, and systemic inflammation, all of which are common among patients at highest risk of severe RSV; an isolated elevation therefore has limited specificity and is unlikely to outperform established clinical predictors [35]. In addition, almost all available RSV severity data derive from infants, in whom baseline D-dimer distributions, thrombotic risk, and disease mechanism differ substantially from the older adults who account for most severe adult RSV disease; the two populations should not be pooled. The greatest potential value of D-dimer may still lie within multimarker prognostic models that integrate inflammatory biomarkers, clinical severity scores, and radiological findings, but this remains untested in RSV. On current evidence, D-dimer should not be described as a promising prognostic biomarker in RSV; it should be described as unevaluated in adults and negative in the single paediatric study available [28].

### 3.4. Middle East Respiratory Syndrome (MERS)

The evidence base for MERS is even more limited, but the available comparative coronavirus literature suggests that coagulation abnormalities were recognized before COVID-19 and were not biologically unprecedented. Reviews comparing coronavirus infections describe elevated D-dimer, thrombocytopenia, prolonged clotting times, and thrombotic complications as part of the coagulation disorder spectrum observed across severe coronavirus disease, including MERS [26]. Pathologically, COVID-19 also shares important features with SARS and MERS, particularly diffuse alveolar damage and severe inflammatory lung injury, which offers a plausible substrate for similar fibrin turnover [13]. We identified no MERS-CoV study reporting an association between D-dimer and a clinical outcome; the available descriptions are of coagulation abnormalities within case series, not of prognostic performance.

Even so, the contrast with COVID-19 is striking. MERS never generated the large, outcome-linked biomarker datasets needed to determine whether D-dimer independently predicts ICU admission, thrombosis, or death, or whether it merely accompanies severe critical illness. Thus, although MERS supports the concept that coronavirus infections can perturb coagulation, it does not support confident pathogen-specific clinical interpretation of D-dimer. The implication is methodological as much as biological: COVID-19 did not reveal an entirely new phenomenon, but it did uniquely permit its systematic quantification.

### 3.5. Other Respiratory Viruses (Adenovirus, Parainfluenza, Human Metapneumovirus)

For adenovirus, parainfluenza, and human metapneumovirus, the literature remains sparse and largely indirect. Across the available evidence, the most defensible synthesis is that severe viral respiratory infections can converge on common final pathways of endothelial activation, inflammation, fibrin formation, and fibrinolysis, so D-dimer elevation is biologically plausible across these pathogens. What is missing is not a mechanism, but rigorous prognostic mapping. The three pathogens are nonetheless not equivalent, and grouping them obscures a real difference in how much is known.

Adenovirus has the strongest of these weak evidence bases. In a retrospective cohort of 699 children with adenovirus pneumonia, median admission D-dimer was 802 ng/mL in severe disease versus 222 ng/mL in non-severe disease; D-dimer remained an independent risk factor for severe pneumonia (adjusted odds ratio 1.005 per ng/mL) within a five-variable model that also included neutrophil percentage, fibrinogen degradation products, B-cell percentage, and lactate dehydrogenase [29]. The reported model discrimination was high (AUC 0.974), but the model was derived and evaluated in a single centre without external validation, D-dimer was not assessed as a stand-alone predictor, and the population was paediatric; the result therefore establishes an adjusted association rather than clinical utility. For human metapneumovirus, the position is different again: even intensive care series of severe hMPV community-acquired pneumonia have reported procalcitonin and C-reactive protein without reporting coagulation markers at all [30]. For parainfluenza, we identified no study relating D-dimer to any clinical outcome.

The current evidence does not establish whether D-dimer in these infections predicts discrete thrombotic complications, general disease severity, or both. This matters because D-dimer performs best when embedded in a disease model with a strong thrombo-inflammatory component and frequent clinically verified thrombosis, as in COVID-19. Where thrombosis ascertainment is inconsistent and cohort sizes are small, D-dimer risks becoming a nonspecific correlate of critical illness rather than a clinically actionable biomarker [13,18].

A common pattern nonetheless emerges across non-COVID-19 viruses: evidence of coagulation activation exists, but prognostic specificity is uncertain. The major knowledge gaps are straightforward: few prospective cohorts, little assay standardization, almost no validated outcome thresholds, and minimal data showing added value beyond conventional clinical indicators. The distinction between kinds of gap matters for how the field should describe itself: for adenovirus the evidence is preliminary, for RSV it is preliminary and negative, and for MERS-CoV, parainfluenza, and human metapneumovirus it is absent. Figure 1 maps this unevenness across viruses and across levels of claim.

## 4. Prognostic Value of D-Dimer

### 4.1. Prediction of Disease Severity

Across viral respiratory infections, the strongest evidence for D-dimer concerns prediction of disease severity, but this conclusion rests overwhelmingly on COVID-19 data. Large meta-analyses consistently show higher D-dimer concentrations in severe versus non-severe COVID-19 and support an independent association with disease progression, with pooled discrimination that is useful but not definitive [10,17,18]. This places D-dimer in the category of a moderately informative severity biomarker rather than a disease-defining test. These are level (i) and level (ii) claims in the hierarchy set out in Section 1; the level (iii) claim, that D-dimer adds discrimination beyond age, oxygenation, and comorbidity, has rarely been tested even in COVID-19, and we found no test of it in any other respiratory virus.

The main interpretive challenge is that severity prediction and pathobiological specificity are not the same. D-dimer rises in response to fibrin turnover, but in severe viral pneumonia, fibrin turnover can reflect macrothrombosis, microvascular thrombosis, endothelial injury, diffuse alveolar damage, or generalized inflammatory organ dysfunction. The strong correlation of D-dimer with other inflammatory and coagulation markers in COVID-19 supports viewing it as a composite marker of thrombo-inflammatory stress rather than a pure thrombosis signal [16,45].

This distinction becomes even more important outside COVID-19. Influenza may show similarly elevated D-dimer levels in hospitalized patients, yet the evidence that D-dimer independently improves severity prediction is much weaker [19]. For RSV, MERS, adenovirus, parainfluenza, and human metapneumovirus, the problem is not contradictory evidence, but insufficient evidence. COVID-19 findings therefore provide a mechanistic template, not a validated universal rule. Table 1 and Figure 1 summarize where each virus currently sits.

### 4.2. Prediction of ICU Admission

Evidence for ICU admission prediction is moderately strong in COVID-19 and weak elsewhere. Early pooled analyses linked elevated D-dimer to composite endpoints that included ICU care, invasive ventilation, and ARDS, while subsequent studies found higher values in hospitalized and severe groups and identified D-dimer as an independent predictor of worse in-hospital trajectories [50,51,52]. Still, ICU admission is a partly system-dependent outcome, influenced by local triage thresholds, bed availability, and institutional practice, which makes cross-study threshold comparisons inherently unstable.

That instability helps explain why universal cutoffs have failed to emerge. Proposed thresholds vary from values near the upper limit of normal to 1.5–2.5 mg/L or higher, depending on whether the endpoint is severity, ICU transfer, thrombosis, or death [5,13,16]. Age, malignancy, chronic inflammation, pregnancy, and comorbidity burden further shift baseline D-dimer distributions, so a threshold that enriches risk in one hospitalized population may overcall risk in another [5,49].

### 4.3. Prediction of Thrombotic Complications

For thrombotic complications, D-dimer has a different performance profile: high sensitivity, but modest specificity. In COVID-19, this makes biological and clinical sense because thrombosis rates were substantial, particularly in critical illness, and D-dimer values were often strikingly elevated in those with coagulation-related complications [15,16]. Meta-analytic diagnostic modeling found sensitivity around 90% but specificity around 60% for COVID-19-related VTE, supporting D-dimer as a screening or rule-out adjunct rather than a confirmatory marker [18].

This outcome domain is where COVID-19 is least transferable to other viruses. The prognostic utility of D-dimer for thrombosis depends on the background incidence and ascertainment of thrombosis within each infection. COVID-19 cohorts actively searched for pulmonary embolism and other thrombotic events because the syndrome raised early concern; comparable surveillance was far less systematic in influenza, RSV, or MERS. A biomarker cannot demonstrate pathogen-specific thrombotic prediction where the outcome itself was inconsistently sought.

### 4.4. Prediction of Mortality

Mortality is the outcome for which D-dimer shows the most clinically recognizable association across the literature, again driven mainly by COVID-19. Multiple studies and meta-analyses report markedly higher D-dimer levels in non-survivors, with adjusted models showing persistent associations after accounting for age and other factors [5,13,17]. Nevertheless, the effect should not be overstated: prognostic accuracy is moderate, and elevated D-dimer identifies risk enrichment, not inevitable death [10,18].

The lack of universal mortality cutoffs reflects both analytical and biological heterogeneity. Assays differ, units differ, the relevant timepoint differs, and mortality itself may be driven by thrombosis in some patients and by overwhelming inflammatory or multiorgan failure in others [5,18]. D-dimer should therefore be interpreted as a broad marker of adverse host response whose prognostic value emerges most clearly when combined with clinical context and other laboratory measures, not as a pathogen-agnostic standalone trigger.

Overall, D-dimer has genuine prognostic value across severe viral respiratory infections, but its validated clinical utility remains largely a COVID-19 achievement rather than a settled cross-viral principle. Expressed in the terms used throughout this review, the field has established association and, for COVID-19, independent association; it has not established incremental value over routine clinical assessment for any virus, and it has not established treatment-predictive value for any virus.

## 5. Clinical Utility and Current Limitations

### 5.1. Interpretation of Elevated D-Dimer

In viral respiratory infections, elevated D-dimer appears to reflect a composite signal of fibrin formation and degradation, systemic inflammation, endothelial injury, and illness severity rather than a single pathophysiologic process. In COVID-19, large meta-analyses consistently show that higher admission D-dimer is associated with severe disease, disease progression, and mortality, supporting its value as a prognostic biomarker but not establishing causality or mechanistic specificity [10,53].

The strongest COVID-19 data therefore support D-dimer as a marker of adverse host response rather than a direct surrogate for macrovascular thrombosis. This distinction is important because D-dimer correlated with inflammatory markers such as hsCRP in COVID-19, and showed only weak correlation with VTE risk scoring in one comparative pneumonia analysis, arguing against interpreting an isolated elevation as evidence of active thromboembolism [40]. That interpretation is also reinforced by studies in which pulmonary embolism and deep vein thrombosis were excluded in patients with high thrombosis probability, yet elevated D-dimer still tracked with mortality and radiographic severity [13].

Outside COVID-19, the evidence base is thinner and less standardized, but the same broad pattern appears to hold. Reviews of coronavirus infections describe elevated D-dimer across COVID-19, SARS-CoV-1, and MERS in the setting of severe systemic disease and thrombo-inflammatory complications [26]. Comparative work also suggests that inflammatory biomarker profiles differ between COVID-19 and influenza A, indicating that D-dimer should not be interpreted identically across viruses [21]. Data for RSV remain scarce and, where directly tested in bronchiolitis, negative [27], and recent paediatric influenza-associated encephalopathy data suggest prognostic value for D-dimer in severe extrapulmonary influenza syndromes, but these findings remain preliminary and cannot yet support broad cross-virus generalization [22].

### 5.2. Variability in Cut-Off Values

A major limitation is the wide variability in proposed prognostic thresholds. In COVID-19 alone, studies have reported admission cut-offs of 1.0, 1.5, 2.0, and 2.14 mg/L or μg/mL-equivalent frameworks, with materially different sensitivities and specificities, while meta-analysis has concluded that several candidate values may be clinically useful rather than identifying one universally valid threshold [4,5,13,53].

This heterogeneity is not surprising. It reflects differences in assay platform, reporting unit, FEU versus DDU calibration, laboratory reference range, sampling time, case mix, disease severity, and exclusion of comorbid conditions known to elevate D-dimer [18,54]. Even when harmonization is attempted across centres, prognostic discrimination remains only modest, as shown in a large Spanish registry in which harmonized admission D-dimer independently predicted mortality but yielded an AUC of only 0.66 [14].

These findings argue against a universal cut-off applicable across viruses, institutions, and assays. Recent international quality-assurance data show that D-dimer assays remain neither standardized nor interchangeable, with particularly high interlaboratory variability around the common 0.5 mg/L FEU threshold used for VTE exclusion [55]. Prognostic interpretation therefore likely requires virus-specific, assay-specific, and possibly time-specific thresholds rather than a single transferable value.

### 5.3. Influence of Age and Comorbidities

Age and comorbidity substantially complicate D-dimer interpretation. In COVID-19 cohorts, patients with higher D-dimer were older and had more underlying disease, and meta-analytic heterogeneity analyses identified age, comorbidity burden, and adjustment strategy as important sources of between-study variation [4,10]. This matters because many chronic conditions independently raise D-dimer, including cardiovascular disease, chronic kidney disease, cancer, and other inflammatory states [56,57].

The confounding effect is especially relevant in older adults and patients with renal dysfunction. D-dimer rises with age in chronic hemodialysis populations and is independently associated with aging-related biomarker change, while elderly patients with chronic heart failure show adverse prognosis above a noninfectious threshold of 885 ng/mL, illustrating how baseline elevation may predate viral infection [57,58]. These observations support individualized or age-aware interpretation, but direct evidence validating age-adjusted prognostic thresholds in viral respiratory infections remains limited.

### 5.4. Challenges in Routine Clinical Application

In routine practice, D-dimer is attractive because it is inexpensive, widely available, and rapidly measured, but these operational advantages are offset by limited specificity and problematic reproducibility across assays [5,17,55]. The literature therefore supports its use primarily for risk stratification, not as a standalone decision trigger. Even meta-analytic estimates describe only moderate prognostic accuracy, and dynamic changes may be more informative than a single baseline value in some settings [18].

A further challenge is translation from association to action. Elevated D-dimer identifies higher-risk patients, but current studies do not define a uniform response to that information, and anticoagulation decisions may themselves confound prognostic analyses [10,18]. For this reason, D-dimer appears most clinically useful when integrated with age, comorbidities, imaging, organ dysfunction, and complementary inflammatory markers rather than interpreted in isolation [21].

Overall, elevated D-dimer is a reproducible marker of adverse prognosis in COVID-19 and a biologically plausible one in other viral respiratory infections, but outside COVID-19, the claim of consistency across viral respiratory infections is not supported by the available clinical data. Its clinical value depends on assay context, timing, baseline patient characteristics, and multimarker interpretation rather than isolated measurement alone.

## 6. Unanswered D-Dimer Research Directions

### 6.1. Is D-Dimer a Marker of Thrombosis or Inflammation?

The central unresolved issue is that D-dimer is biologically specific for fibrin degradation, but clinically nonspecific for the process that generated that fibrin. In COVID-19, elevated values are compatible with macrovascular thrombosis, pulmonary microthrombosis, endothelial injury, and systemic inflammatory activation, yet retrospective cohorts rarely adjudicated venous thromboembolism systematically and therefore could not separate clot burden from generalized illness severity [13].

Mechanistic studies support a broader thrombo-inflammatory interpretation. Meta-regression data link D-dimer to inflammatory cytokines and organ-injury markers, while pathology-based reviews emphasize endotheliitis, megakaryocyte-rich microvascular injury, and dysregulated fibrinolysis rather than classic consumptive coagulopathy alone [59,60]. At the same time, viscoelastic studies suggesting “fibrinolysis shutdown” highlight an apparent paradox: high circulating D-dimer can coexist with impaired whole-blood clot lysis, implying that compartmentalized pulmonary fibrin turnover may not be captured by conventional thrombosis models [61]. Progress will require prospective studies combining serial D-dimer with imaging-confirmed thrombosis, endothelial biomarkers, viscoelastic assays, and where feasible, bronchoalveolar or autopsy correlates.

### 6.2. Why Does Prognostic Performance Differ Among Respiratory Viruses?

The literature suggests that prognostic performance differs across viruses, but it remains unclear how much of that variation is biological and how much is methodological. COVID-19 has the strongest evidence base, whereas influenza, RSV, MERS, adenovirus, and human metapneumovirus are represented largely by smaller or indirect studies, making between-virus comparisons inherently unstable [13,62].

Available comparative data do not support a simple “higher is worse only in COVID-19” model. In one direct comparison, D-dimer levels were similarly elevated in COVID-19 and seasonal influenza despite more marked platelet-driven coagulopathy in COVID-19, suggesting that prognostic differences may reflect distinct host responses and coagulation architectures rather than absolute D-dimer concentration alone [19]. Earlier reviews also note that coagulation dysregulation contributes to influenza pathogenesis, reinforcing the plausibility of shared but nonidentical thrombo-inflammatory pathways across respiratory viruses [13]. Priority research should therefore use harmonized assays and parallel enrollment across multiple viral pneumonias to determine whether D-dimer adds unique prognostic value beyond baseline severity within each pathogen.

We considered whether an independent comparative analysis of publicly available datasets could be incorporated into this review, and concluded that it could not be done responsibly at present, for three reasons. First, D-dimer is reported in incompatible units and calibrations across cohorts, including fibrinogen equivalent units versus D-dimer units, so values cannot be pooled without assay-level metadata that most repositories do not provide [54,55]. Second, the non-COVID-19 cohorts that record D-dimer at all are small, so any cross-virus comparison would be underpowered for the adjusted analyses that would make it informative. Third, thrombosis was ascertained by different strategies in each cohort, which biases comparison of the outcome itself rather than only of the biomarker. The materials to begin such work nonetheless exist—for example, patient-level intensive care data comparing D-dimer across influenza A pneumonia, COVID-19, septic shock, and pulmonary embolism are openly deposited [20]—and in our view, a federated analysis across harmonized repositories, reporting assay and units for every contributing cohort, is the single most valuable next study in this field. We therefore flag it as a specific and feasible research priority rather than attempting an underpowered version of it here.

### 6.3. Can D-Dimer Guide Anticoagulation Strategies?

D-dimer-guided anticoagulation remains appealing but unproven. Observational studies associated rising first-week D-dimer with death and thrombosis, and some institutional anticoagulation intensification coincided with lower D-dimer trajectories and fewer adverse events, but these designs are highly vulnerable to confounding by indication, secular changes in care, and selective testing [63].

Randomized evidence has not established D-dimer as a reliable treatment-selection biomarker. In action, therapeutic anticoagulation in hospitalized patients with elevated D-dimer did not improve clinical outcomes and increased bleeding [31]. By contrast, the multiplatform heparin trial showed benefit in noncritically ill patients, with high posterior probabilities of superiority across D-dimer strata rather than an exclusive effect in one threshold-defined subgroup [32]. Smaller D-dimer-driven trials also failed to show clear advantage in critically ill patients or with rivaroxaban-based strategies [33,34]. The unresolved question is therefore not whether D-dimer identifies risk, but whether it identifies modifiability of risk. Future trials should randomize on dynamic trajectories, not single baseline values, and should distinguish heparin from direct oral anticoagulants and ward from ICU populations. No randomized trial has tested a D-dimer-guided anticoagulation strategy in any non-COVID-19 respiratory viral infection.

### 6.4. Need for Standardized Thresholds

The variability of reported thresholds is described in Section 5.2 and is not repeated here; the point relevant to future work is that reported prognostic cut-offs in COVID-19 have ranged from roughly 0.4 to more than 3.0 mg/L, reflecting differences in assay platforms, reporting units, patient age, disease stage, and clinical endpoint definition rather than true biological precision [5,64,65].

This problem begins with laboratory heterogeneity. Many studies did not report the assay manufacturer, did not distinguish between D-dimer units (DDUs) and fibrinogen equivalent units (FEUs), and did not provide assay-specific reference ranges, making direct comparison of D-dimer concentrations across studies difficult and sometimes misleading [54].

Even in PE-oriented emergency cohorts, age-adjusted thresholds modestly improved specificity while performance was somewhat lower in SARS-CoV-2-positive patients, arguing against a single universal cut point for all settings [66]. A realistic next step is harmonization of units and reporting standards, followed by derivation of disease-specific and context-specific thresholds that can later be individualized by age, comorbidity burden, and illness phase.

### 6.5. Emerging Multimarker and Precision Medicine Approaches

D-dimer alone is unlikely to support sufficiently precise prognostication because it reflects only one component of a multidimensional syndrome. Systematic reviews and cohort studies show that CRP, ferritin, LDH, troponin, platelet count, and clinical severity measures each capture complementary information about inflammation, tissue injury, and host response [62,65].

Multimarker models already appear to outperform isolated biomarkers in adjacent respiratory infection settings. In paediatric severe mycoplasma pneumonia, D-dimer combined with inflammatory mediators and LDH improved severity discrimination, illustrating how coagulation markers may gain value when embedded in broader host-response panels rather than interpreted in isolation [67]. The future agenda should therefore prioritize externally validated multimarker models, serial rather than static measurements, and machine-learning approaches that integrate laboratory, physiologic, and virologic data without obscuring clinical interpretability. Across viral respiratory infections, the key gap is no longer whether D-dimer is associated with poor outcomes, but when, in whom, and in combination with which other signals it can improve individualized management.

## 7. Conclusions and Future Perspectives

D-dimer has emerged as one of the most extensively investigated biomarkers of adverse outcomes in viral respiratory infections, particularly during the COVID-19 pandemic. In COVID-19, the evidence supports a reproducible and independent association between elevated D-dimer and disease severity, intensive care admission, thromboembolic complications and mortality, and this has established D-dimer as a component of clinical risk stratification. It does not establish that D-dimer adds information beyond routine clinical assessment, and randomized trials have not shown that D-dimer identifies patients who benefit from escalated anticoagulation. Beyond SARS-CoV-2, the evidence is not merely thinner but qualitatively different: influenza is supported chiefly by comparative cohorts, adenovirus by a single unvalidated paediatric model, bronchiolitis by one negative paediatric study, and MERS-CoV, parainfluenza, and human metapneumovirus by no outcome-linked data at all. Consequently, the prognostic significance of D-dimer beyond COVID-19 cannot yet be considered established, and the cross-viral inferences drawn in this review should be read as hypotheses rather than conclusions.

Current evidence suggests that D-dimer should be interpreted as an integrated biomarker reflecting the complex interplay between coagulation activation, fibrin turnover, endothelial injury, and systemic inflammation rather than as a specific indicator of thrombotic events alone. Its clinical value is therefore maximized when interpreted alongside patient characteristics, disease severity, imaging findings, and complementary laboratory markers instead of being used in isolation. Variability in assay methodology, reporting units, sampling time, and patient populations continues to limit the establishment of universally applicable prognostic thresholds and complicates comparisons across studies and viral pathogens.

Future research should prioritize large, prospective, multicentre studies that directly compare major respiratory viruses using standardized D-dimer assays and harmonized clinical outcome definitions. Serial measurements should be incorporated to determine whether dynamic changes provide greater prognostic value than single baseline assessments. Integrating D-dimer with biomarkers of endothelial injury, inflammation, cardiac dysfunction, and immune activation may facilitate the development of robust multimarker prediction models capable of supporting individualized clinical decision-making. Furthermore, mechanistic studies are needed to clarify the relative contributions of thrombosis, immunothrombosis, and inflammatory lung injury to D-dimer elevation across different viral infections. Three specific and testable propositions follow from this review, each addressable with existing methods: first, that the apparently weaker prognostic performance of D-dimer outside COVID-19 is driven largely by lower thrombotic event rates and less systematic thrombosis ascertainment rather than by fundamentally different biology; second, that change in D-dimer over the first 72 h carries more prognostic information than any single admission value in any severe viral pneumonia; and third, that D-dimer adds little or no discrimination to routine severity variables when formally tested, in COVID-19 as well as elsewhere. Ultimately, translating biological plausibility into clinically actionable evidence will require standardized methodologies, external validation across diverse populations, and precision medicine approaches that define when, in whom, and how D-dimer can meaningfully improve patient management beyond COVID-19.

## Figures and Tables

**Figure 1:**
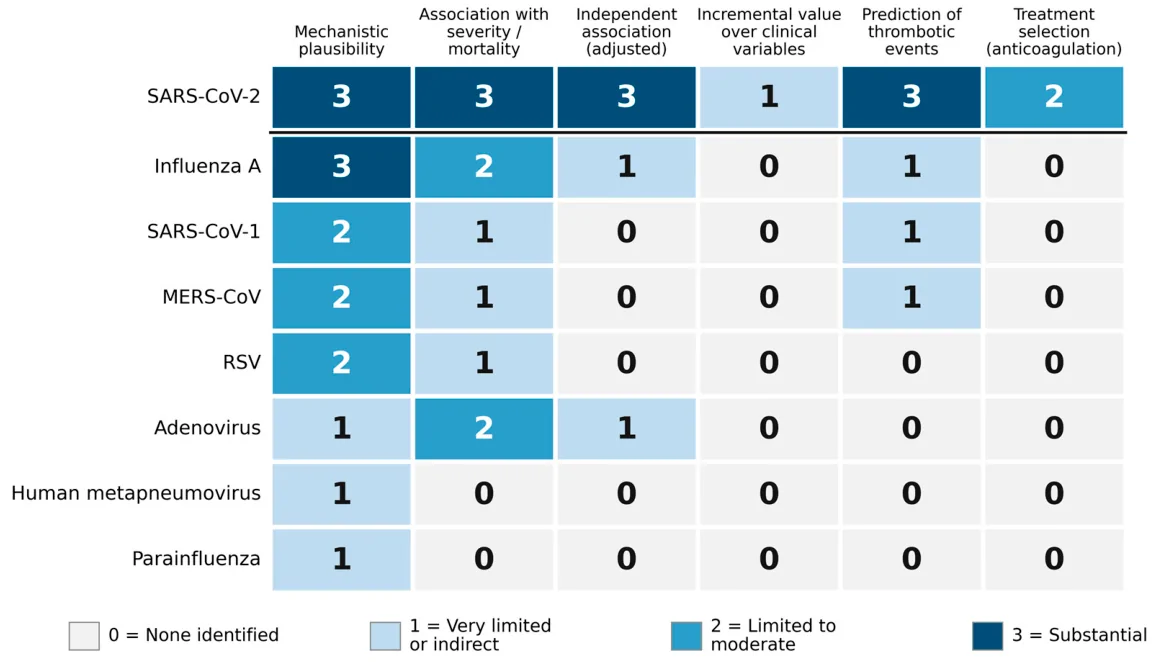
Evidence map for D-dimer across respiratory viruses and evidence domains. Cells are graded 0–3 for the strength of available human clinical evidence, not for the direction of the finding: 0, none identified; 1, very limited or indirect; 2, limited to moderate; 3, substantial. The studies in this review that support each non-zero grade are as follows. SARS-CoV-2: mechanistic plausibility [6,7,8,9]; association with severity/mortality [4,10,13,17]; independent (adjusted) association [4,14]; incremental value over clinical variables [14]; prediction of thrombotic events [15,16,18]; treatment selection (anticoagulation) [31,32,33,34]. Influenza A: mechanistic plausibility [8,11]; association with severity/mortality [19,20,21,22]; independent association [22]; prediction of thrombotic events [19,23]. SARS-CoV-1 and MERS-CoV (each): mechanistic plausibility [11,26]; association with severity/mortality [26]; prediction of thrombotic events [26]. RSV: mechanistic plausibility [11,35]; association with severity/mortality [27]. Adenovirus: mechanistic plausibility [11]; association with severity/mortality [29]; independent association [29]. Human metapneumovirus and parainfluenza (each): mechanistic plausibility [11]. Cells graded 0 carry no reference because no supporting study was identified. Two features are apparent. First, within every virus the domains on the right, incremental value over routine clinical variables and treatment selection, are far less well supported than simple association, and this holds for SARS-CoV-2 (shown above the rule as the reference pathogen) as well as for the others. Second, evidence weakens from top to bottom, so that mechanistic plausibility is the only domain populated for most non-COVID-19 viruses. Three grades warrant comment: the grade of 2 for treatment selection in SARS-CoV-2 reflects the existence of randomized trials, several of which were neutral or negative [31,32,33,34], and not evidence of benefit; the grade of 1 for prediction of thrombotic events in influenza rests on indirect coagulopathy data [19,23], because the two large cohorts quantifying thrombotic incidence in influenza did not themselves measure D-dimer [24,25]; and the mechanistic grade of 1 for human metapneumovirus and parainfluenza rests on the general immunothrombosis literature rather than pathogen-specific data [11].

**Figure 2:**
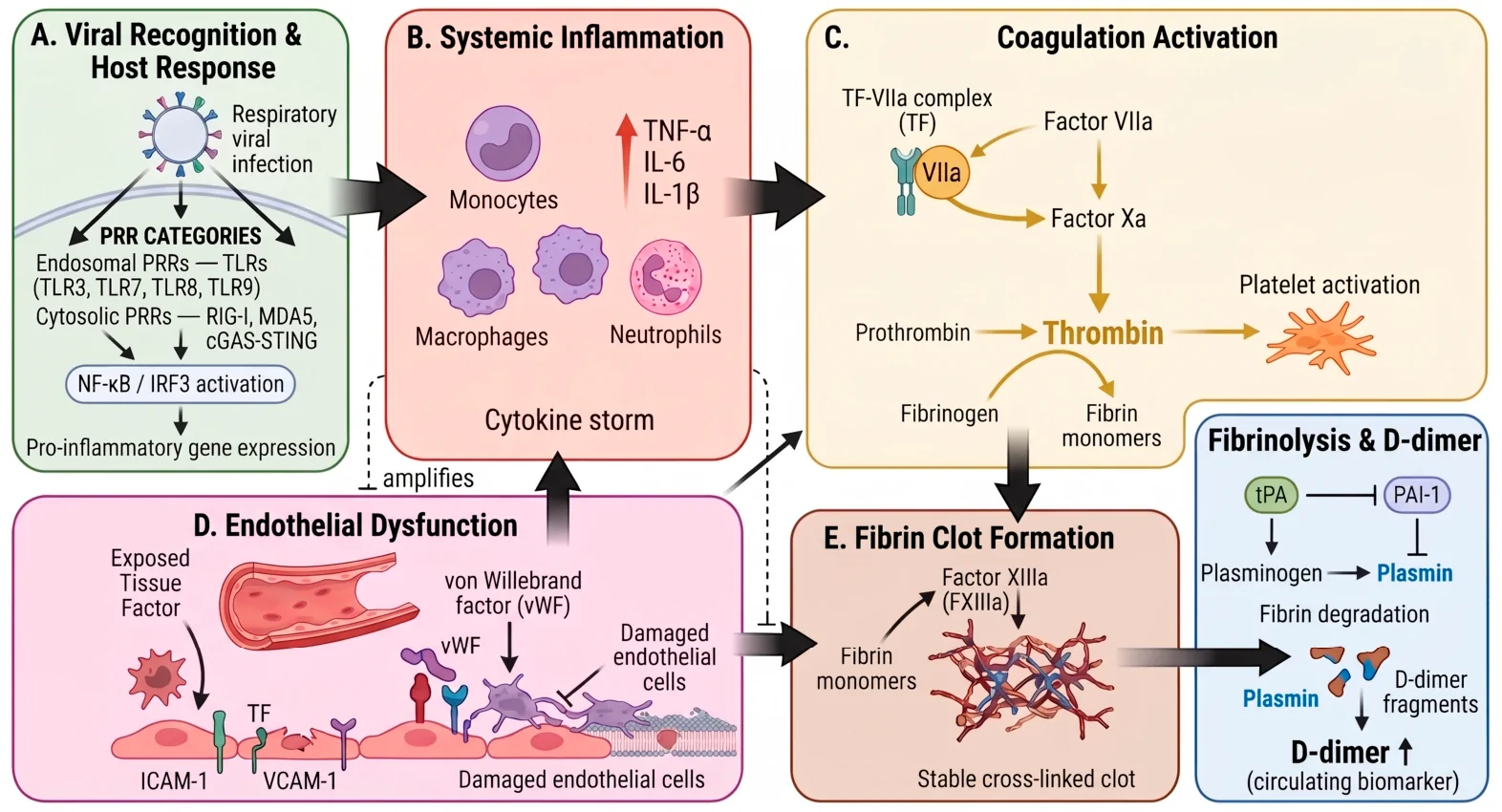
Mechanistic cascade linking viral recognition to endothelial dysfunction, hypercoagulability, and fibrinolysis. (**A**) Viral recognition and host response. Respiratory viral entry triggers endosomal (TLR3, TLR7, TLR8, TLR9) and cytosolic (RIG-I, MDA5, cGAS-STING) pattern recognition receptors (PRRs), signaling via NF-κB and IRF3 activation to induce pro-inflammatory gene expression. (**B**) Systemic inflammation. Upregulated cytokines (TNF-α, IL-6, IL-β) drive a cytokine storm alongside monocyte, macrophage, and neutrophil mobilization. (**C**) Coagulation activation. Inflammation initiates the extrinsic pathway via Tissue Factor (TF)–Factor VIIa complex formation, downstream Factor Xa activation, and thrombin generation from prothrombin. Thrombin cleaves fibrinogen into fibrin monomers and drives platelet activation. (**D**) Endothelial dysfunction. Endothelial damage leads to exposed TF, adhesion molecule expression (ICAM-1, VCAM-1), and von Willebrand factor (vWF) release, establishing a feedback loop that amplifies systemic inflammation. (**E**) Fibrin clot formation. Factor XIIIa (FXIIIa) cross-links fibrin monomers into a stable fibrin meshwork (Fibrinolysis and D-dimer). Tissue plasminogen activator (tPA)—regulated by plasminogen activator inhibitor-1 (PAI-1)—converts plasminogen to plasmin to catalyze fibrin degradation, resulting in elevated circulating D-dimer levels. The image was generated using ImageGen v2.0.

**Table 1:** Clinical evidence linking D-dimer to outcome, by respiratory virus. Every primary study is cited where it is first described in the “Studies identified” column, and each remaining cell carries the reference for the specific finding it reports, so that no statement in the table is unsourced; no cell draws on studies not listed in that row. Study design is stated so that primary clinical studies can be distinguished from reviews and meta-analyses. An em dash indicates that the item was not assessed because no study was identified.

Virus	Studies Identified (Design)	Typical D-Dimer Finding	Outcomes Assessed	Adjusted/Independent Association	Threshold and Discrimination	Strength of Prognostic Evidence
SARS-CoV-2	Numerous. Used here: an admission cohort [4] and a case-control study [13]; a multicentre harmonized registry [14]; a multicentre prospective cohort of thrombosis in severe disease [15] and a hospital cohort of thrombotic and bleeding manifestations [16]; systematic reviews and meta-analyses [10,17,18]	Higher on admission and at peak in severe disease and in non-survivors	Severity, ICU admission, VTE, in-hospital mortality	Yes; independent predictor of mortality in adjusted models [4,14]	Admission cut-offs vary by study (e.g., 2.0 mg/L) [4]; AUC 0.66 with harmonized assays [14]; pooled sensitivity ~90% and specificity ~60% for VTE [18]	Substantial for association; moderate for discrimination; rarely tested for incremental value
Influenza A	Few. One comparative cohort of hospitalized influenza versus COVID-19 [19]; one prospective ICU study across four critical illnesses [20]; one comparative prognostic-marker study [21]; one paediatric influenza-associated encephalopathy cohort [22]; one conference abstract on thrombotic biomarkers [23]; two large cohorts quantifying thrombotic event rates that did not measure D-dimer [24,25]	Comparable to COVID-19 in hospitalized patients [19]; elevated in critically ill influenza A pneumonia but not virus-specific [20]	Severity and mortality; prognosis in influenza-associated encephalopathy; thrombotic event incidence	Not established in influenza pneumonia; a single paediatric encephalopathy cohort reports a prognostic association [22]	None validated; no influenza-specific threshold proposed	Limited and largely indirect
SARS-CoV-1	None identified. Coagulation abnormalities described only within comparative reviews [11,26]	Elevated D-dimer described qualitatively in severe disease [26]	Descriptive only	No	None	Very limited
MERS-CoV	None identified. Coagulation abnormalities described only within comparative reviews [11,26]	Elevated D-dimer described within a coagulation disorder spectrum [26]	Descriptive only	No	None	Very limited
RSV	One retrospective paediatric cohort of hospitalized bronchiolitis, n = 102 [27]. No adult RSV study identified; a current RSV review reports no D-dimer outcome data [28]	No difference in serum D-dimer between severity groups [27]	Clinical severity score, length of stay, PICU admission; no deaths occurred [27]	No association detected	None	Very limited; the single direct test was negative
Adenovirus	One retrospective paediatric cohort of adenovirus pneumonia, n = 699 [29]	Median admission D-dimer 802 versus 222 ng/mL in severe versus non-severe disease [29]	Severe adenovirus pneumonia	Yes; aOR 1.005 per ng/mL within a five-variable model [29]	No stand-alone threshold; five-variable model AUC 0.974, not externally validated [29]	Limited; single centre, paediatric, D-dimer not evaluated alone
Human metapneumovirus	None relating D-dimer to outcome. A ten-year ICU series of severe hMPV pneumonia reported procalcitonin and CRP but no coagulation markers [30]	Not reported	—	—	—	None
Parainfluenza	None identified	Not reported	—	—	—	None

VTE, venous thromboembolism; PICU, paediatric intensive care unit; CRP, C-reactive protein; aOR, adjusted odds ratio; AUC, area under the receiver operating characteristic curve.

## Data Availability

Figure 1 and Figure 2 were drawn by the authors; AI-assisted tools were used for language editing only. All scientific concepts, interpretations, and conclusions are the original work of the authors. No new data were created or analysed in this study.

## Supplementary Materials

The following supporting information can be downloaded at: https://www.mdpi.com/article/10.3390/ijms27198525/s1.
