# Supplementary material for: D-Dimer in Viral Respiratory Infections: Established Evidence in COVID-19 and Emerging Evidence in Other Respiratory Viruses
Source: Int J Mol Sci. 2026 Sep 24;27(19):8525. doi: 10.3390/ijms27198525 (PMC13664812; doi:10.3390/ijms27198525)
Supplement: Supplementary file 1 [file ijms-27-08525-s001.zip › ijms-4516372-supplementary.pdf]

Supplementary Table S1. Database search strings by pathogen.

Searches covered articles published from 1 January 2000 to 20 August 2026, the date on which the final search was run, and were limited to English-language full-text reports. For every pathogen, the common biomarker block (Block A) was combined with the pathogen-specific block (Block B) using AND. Reference lists of retrieved articles were hand-searched for further eligible studies. The number of eligible primary studies retained for each pathogen is reported in Table 1 of the main text.

Block A — common biomarker block

PubMed/MEDLINE: ("D-dimer"[tiab] OR "D dimer"[tiab] OR "Fibrin Fibrinogen Degradation Products"[Mesh]) AND ("Blood Coagulation Disorders"[Mesh] OR "coagulopathy"[tiab] OR "immunothrombosis"[tiab] OR "Prognosis"[Mesh] OR "Severity of Illness Index"[Mesh] OR "Mortality"[Mesh] OR "Biomarkers"[Mesh])

Filters applied: English[lang] AND 2000/01/01:2026/08/20[dp]

Scopus: TITLE-ABS-KEY("D-dimer" OR "D dimer") AND TITLE-ABS-KEY(coagulopathy OR immunothrombosis OR prognosis OR severity OR mortality OR biomarker) AND PUBYEAR > 1999 AND LANGUAGE(english)

Google Scholar: "D-dimer" AND (prognosis OR severity OR mortality) AND <pathogen term>; first 100 results screened by title and abstract.

Block B — pathogen-specific blocks

Pathogen	PubMed/MEDLINE	Scopus
SARS-CoV-2	"COVID-19"[Mesh] OR "SARS-CoV-2"[Mesh] OR "COVID-19"[tiab] OR "SARS-CoV-2"[tiab]	TITLE-ABS-KEY("COVID-19" OR "SARS-CoV-2")
Influenza A	"Influenza, Human"[Mesh] OR "Influenza A virus"[Mesh] OR "influenza"[tiab] OR "H1N1"[tiab] OR "H3N2"[tiab]	TITLE-ABS-KEY(influenza OR "H1N1" OR "H3N2")
SARS-CoV-1	"Severe Acute Respiratory Syndrome"[Mesh] OR "SARS Virus"[Mesh] OR "SARS-CoV-1"[tiab]	TITLE-ABS-KEY("SARS" OR "SARS-CoV-1")
MERS-CoV	"Middle East Respiratory Syndrome Coronavirus"[Mesh] OR "MERS"[tiab] OR "Middle East respiratory syndrome"[tiab]	TITLE-ABS-KEY("MERS" OR "Middle East respiratory syndrome")
RSV	"Respiratory Syncytial Virus Infections"[Mesh] OR "Respiratory Syncytial Viruses"[Mesh] OR "RSV"[tiab] OR "Bronchiolitis"[Mesh]	TITLE-ABS-KEY("respiratory syncytial virus" OR RSV OR bronchiolitis)
Adenovirus	"Adenoviridae Infections"[Mesh] OR "Adenoviruses, Human"[Mesh] OR "adenovirus"[tiab]	TITLE-ABS-KEY(adenovirus)
Human metapneumovirus	"Metapneumovirus"[Mesh] OR "human metapneumovirus"[tiab] OR "hMPV"[tiab]	TITLE-ABS-KEY("human metapneumovirus" OR hMPV)
Parainfluenza	"Respirovirus Infections"[Mesh] OR "Parainfluenza Virus 1, Human"[Mesh] OR "parainfluenza"[tiab] OR "HPIV"[tiab]	TITLE-ABS-KEY(parainfluenza OR HPIV)

Abbreviations. [Mesh], Medical Subject Heading; [tiab], title/abstract field; [dp], date of publication; TITLE-ABS-KEY, Scopus title, abstract and keyword field. D-dimer has no dedicated MeSH heading and is indexed under Fibrin Fibrinogen Degradation Products (unique identifier D005338).
